# Sequential separation of anti-diabetic drugs in the presence of melamine as impurity using chromatographic methods

**DOI:** 10.1186/s13065-025-01385-6

**Published:** 2025-01-28

**Authors:** Maimana A. Magdy, Maha M. Abdelrahman, Doaa G. Mohamed, Amal B. Ahmed

**Affiliations:** 1 Pharmaceutical Analytical Chemistry, Faculty of Pharmacy, Beni-Suef University, Alshaheed Shehata Ahmad Hegazy St, Beni-Suef, 62514 Egypt; 2 Pharmaceutical Chemistry Department, Faculty of Pharmacy, Nahda University, Sharq El-Nile, Beni-Suef, 62511 Egypt

**Keywords:** Saxagliptin, Metformin, Melamine, Ultra- performance liquid chromatography, High- performance thin layer chromatography

## Abstract

The study of green analytical chemistry has garnered significant attention in the context of mitigating global environmental contamination. In this study, we present two methodologies for environmentally friendly chromatography that enable simultaneous and specific determination of Saxagliptin (SAX), metformin (MET), and a pharmacopoeial impurity of MET known as melamine (MEL). The initial method employed in this study is High-Performance Thin Layer Chromatography (HPTLC), which utilized 60 F 254 silica gel-coated Mark HPTLC plates on aluminum sheets as the stationary phase. The developing system was made up of a mix of ethyl acetate: methanol: ammonia: glacial acetic acid in a ratio of 6:4:1:0.3 (v/v/v/v). The analysis was performed at a wavelength of 215 nm. The second method employed in this study is ultra-performance liquid chromatography (UPLC). In this method, a C18 column was utilized for the separation process. The mobile phase was made up of a mix of methanol and 0.01 M sodium dodecyl sulfate, with a pH of 3.3 achieved by adding orthophosphoric acid. The ratio of methanol to sodium dodecyl sulfate in the mobile phase was 70:30 (v/v). The flow rate of the mobile phase was established at a rate of 1.5 mL/min. The peaks found and recorded are resolved at a wavelength of 215 nm. The three analytes under investigation were successfully separated and assessed using the recommended protocols. Both methods were validated following the International Council for Harmonization (ICH) recommendations for assessing linearity, range, accuracy, precision, specificity, and robustness. Moreover, the environmental sustainability of the advanced methodologies The assessment has been performed using various instruments, such as the Analytical Eco-Scale, NEMI, GAPI, and AGREE. The utilization of these tools was implemented in order to perform a comprehensive assessment of the environmental sustainability of the methods, as well as to establish a comparison with previously documented approaches. This study was carried out to evaluate the potential environmental implications of the suggested methods and to determine their suitability for concurrent analysis of the examined pharmaceuticals in formula and quality control units.

**Supplementary Information:**

The online version contains supplementary material available at 10.1186/s13065-025-01385-6.

## Introduction

Saxagliptin (SAX), a non-official medication, SAX HCl is an oral hypoglycemic medicine belonging to the dipeptidyl peptidase-4 (DPP-4) inhibitor family. It is used to treat type II diabetes, either on its own or in conjunction with other medications [1, 2].

Metformin (MET) this drug is categorized as an oral antidiabetic agent that efficiently enhances the responsiveness of insulin in peripheral and hepatic tissues while reducing the likelihood of lactic acidosis. Metformin is frequently prescribed as the first-line medication for persons diagnosed with type-2 diabetes mellitus. Moreover, it has received official recognition in the European Pharmacopoeia [3], Indian Pharmacopeia [4], and United States Pharmacopeia [5]. As a result, the combination of SAX and MET has been widely used to control type II diabetes.

Melamine (MEL) it is considered a related chemical and a likely metformin impurity according to the European Pharmacopoeia [3] and United States Pharmacopeia [5]. MEL poisoning can cause skin, eye, or lung irritation, depending on whether melamine is inhaled or comes into touch with the skin. In addition, prolonged exposure can lead to renal damage, kidney failure, and eventually, mortality [6].

Through a comprehensive analysis of previous academic research, it has been found that the simultaneous determination of SAX and MET as a combination of two substances has been successfully achieved utilizing various analytical methods, such as spectrophotometry [7, 8], RP-HPLC [9–21], and UPLC procedures [22]. The presence of MET and its impurity was assessed using the HPLC Method [23]. In addition, many chromatographic methods [24, 25] were used to assess the presence of MEL, which is the main impurity of MET.

Detecting pharmaceutical impurities is a top priority in pharmaceutical quality control, particularly when such impurities pose a health risk. Consequently, determining the maximum detectable level (MEL) of a pharmacopeial impurity is crucial for making an educated approximation as to the maximum detectable amount (MET) of the target molecule.

Upon reviewing the existing literature, it is evident that there is a lack of published approaches for estimating SAX, MET, and MEL. The objective of this work is to develop precise, sensitive, and dependable chromatographic methods for simultaneously determining SAX, MET, and MET pharmacopeial impurity (MEL) in both raw materials and pharmaceutical dosage forms. These approaches should demonstrate a considerable degree of sensitivity and selectivity. Furthermore, it is crucial to verify the created techniques in accordance with the regulations established by the ICH (International Council for Harmonization of Technical Requirements for Pharmaceuticals for Human Use) [26]. The importance of green analysis has experienced a significant rise in several industries, including pharmaceutical research. Upon further consideration, it becomes apparent that chromatographic techniques require significant amounts of organic solvents that possess both hazardous and non-biodegradable properties. This work introduces a new method that combines a green high-performance thin-layer chromatography (HPTLC) and ultra-performance liquid chromatography (UPLC) assay technique to accurately measure the levels of Saxagliptin, Metformin, and Melamine simultaneously. In addition, a range of assessment approaches are used to evaluate the environmental sustainability of the established method.

## Experimental

### Instrumental

- For HPTLC-densitometric method

The equipment utilized in this research comprised an HPTLC scanner 3 densitometer (Camag, Muttenz, Switzerland) under the control of WINCATS software (version 3.15). Additionally, a sample applicator for HPTLC Linomat V equipped with a 100 μL syringe (Camag, Muttenz, Switzerland) was employed. Furthermore, an ultraviolet (U.V.) lamp with a short wavelength of 254 nm (VL-6.LC, as described by Marne et al.) was utilized. During the absorbance mode of HPTLC scanning, radiation sources such as HPTLC plates measuring 20*10 cm and a deuterium lamp were utilized. The scanning velocity was configured to 20 mm/s, the width and height of the slit were adjusted to 3 × 0.45 mm.

- For the UPLC method

The UPLC method employed the Dionex UltiMate 3000 instrument (Thermo et al., USA), which consisted of various components, including a pump (HPG-3400), a diode array detector (DAD3000RS), a column compartment (TCC-3100), and a Dionex ultimate 3000 autosampler (WPS-3000). The software was Chromeleon, USA. version 7.2. The separation and quantification process involved the utilization of a Hypersil GOLD C18 column (150 mm × 2.1 mm, 1.7 μm).

### Materials

#### Pure standards

Standard MET was generously donated by Sigma Pharmaceuticals Industries (El Monofeya, Egypt), whose manufacturer certificate asserts a purity level of 99.88%—Standard SAX donated by Novartis Pharma (Cairo, Egypt) with a certified purity of 99.97%. The pure standard MEL was procured from Sigma-Aldrich, Germany, and possessed a confirmed purity level of 99.56%.

#### Pharmaceutical formulation

Kombiglyze® XR tablets batch number (3932500) are labeled to contain 5 mg Saxagliptin HCl, and 1000 mg metformin HCl manufactured by AstraZeneca was purchased from the Egyptian pharmaceutical market.

#### Chemicals and reagents

- Methanol of HPLC, as specified by Tedia et al. The object was acquired from Cornell Lab, located in Cairo, Egypt.
- 33% ammonia, Ethyl acetate, ortho-phosphoric acid and, glacial acetic acid of analytical grade were bought from El-Nasr Pharmaceutical Chemicals Co. (El-Saida et al.), while sodium dodecyl sulfate was obtained from LobaChemie, India.
- Deionized water comes from SEDICO Pharmaceutical Co., which is situated in Cairo, Egypt.

### Standard solution

The stock standard solutions of SAX, MET, and MEL were created at a concentration of (1000 μg/mL). This was achieved by weighing 100.00 mg of each substance into individual 100-mL volumetric flasks, and then fill the volumetric flask with methanol up to its volume. Working standard solutions (100 μg/mL) were prepared by accurately and separately transferring 10 mL of each drug into a 100 mL volumetric flask and completing the volume with methanol.

### Methods

#### Chromatographic conditions

##### HPTLC method

The HPTLC method utilized aluminum sheets of 20 by 10 cm The Merck HPTLC silica gel 60 F254 aluminum sheet were obtained from sigma -Aldrich Chemie Gmbh, Germany. The bands on the HPTLC plates were positioned 5 mm from each other and 10 mm from the lower edge. A mixture of ethyl acetate, methanol, ammonia, and glacial acetic acid (6:4:1:0.3, v/v/v/v) was pre-saturated in a chromatographic tank for 30 min at room temperature before linear ascending development was performed. After development, the plates were left out in the open air to dry before being scanned at 215 nm with the prescribed instrumental settings.

##### UPLC method

The UPLC method employed chromatographic separation on a C18 column (150 mm × 2.1 mm, 1.7 μm) at a temperature of 25 °C. Isocratic elution mode was utilized, the mobile phase employed in this study consisted of a methanol and 0.01 M sodium dodecyl sulfate combination adjusted to pH of 3.3, modified by orthophosphoric acid, in a ratio of 70:30 (v/v). The mobile phase underwent filtration using a 0.45-μm Millipore Durapore® membrane filter. Subsequently, it was administered at a constant flow rate of 1.5 mL/min. The injection volume was 5 µL. The scanning procedure was conducted at a wavelength of 215 nm.

#### Linearity and construction of the calibration curves

##### HPTLC method

A set of 10-mL volumetric flasks were employed to transfer different aliquots, ranging from 2 to 100, 2–100 and 0.2–10% of SAX, MET, and MEL, respectively, from their respective standard stock solutions with a concentration of 1 mg/mL. Subsequently, methanol was introduced until the container reached its maximum capacity. The Merck HPTLC silica gel 60 F254 aluminum sheets were subsequently treated by applying bands of 10 μL of each solution in triplicate. The bands were subjected to scanning at a wavelength of 215 nm.

##### UPLC method

A 10 mL volumetric flask were employed to transfer several dilutions of pure SAX, MET, and MEL. These dilutions spanned concentration ranges of 0.5–100, 0.5–70 and 0.2–10% respectively, derived from their respective working solutions with a concentration of 100 μg/mL. Subsequently, The volumes were completely filled with the mobile phase. For each concentration, injections of 20 μL were administered in this experiment. The injections were administered at a flow rate of 1.5 mL/min, and the resultant effluent was analyzed using UV scanning at a wavelength of 215 nm. The separation was performed using the chromatographic technique. The acquired chromatograms revealed the existence of peak regions that corresponded to SAX, MET, and MEL. The calibration graph for each component was constructed by plotting the peak area as a function of the corresponding concentration.

#### Application to pharmaceutical formulation (kombyglize®XR)

A quantity of 0.1656 g of MET and 3.3 g of SAX were individually introduced into two separate 100-mL volumetric flasks following the precise weighing and pulverization of ten kombyglize® tablets. The solutions underwent sonication for 20 min, following which the volume was subsequently modified to 100 mL using methanol. Subsequently, the solutions were individually subjected to filtration using a 90 mm filter paper to produce stock solutions with concentrations of 1 mg/mL for MET and 100 μg/mL for SAX. The operational formulation of MET was subsequently derived from its corresponding stock solutions, and different concentrations of SAX and MET were prepared by diluting the appropriate solutions with the UPLC mobile phase for the UPLC technique.

## Results and discussion

The chromatographic technique can resolve complicated mixtures in pharmaceutical compounds [27–29]. Different analytical methods for analyzing SAX and MET in their binary combinations have been identified in the literature. However, to date, no chromatographic method has been devised for the simultaneous quantification of SAX and MET in the presence of a hazardous MET impurity (MEL). The creation and demonstration of two high-resolution, high-accuracy, selective chromatographic methods for the combined separation of SAX, MET, and MET impurity (MEL) with high resolution are the focus of this study. Both recommended chromatographic procedures were environmentally friendly and used green organic solvents instead of other nephrotoxic solvents used before [22]. Herein, the suggested HPTLC approach exhausts methanol and ethyl acetate as developing solvents instead of the nephrotoxic chloroform in the published method; therefore, it has the advantage of being more environmentally friendly.

### Method development and optimization

#### HPTLC method

HPTLC-densitometry analysis the initial approach employed for the differentiation and evaluation of the two anti diabetic medicines, as well as the potentially harmful impurity MET (MEL), was investigated. The approach mentioned above demonstrates exceptional levels of sensitivity and selectivity while also providing cost and time efficiency.

- Developing a system

Regarding green analytical chemistry, several tests were performed using various developing systems. Firstly, isopropanol, butanol, and various ratios of ethanol and acetone were used in the experiments, both with and without ammonia, acetic acid and formic acid solution. Regrettably, the examined components exhibited suboptimal resolution. Subsequently, different ratios of ethyl acetate: methanol were inspected; the promising ratio was (6:4, v/v), but MET and MEL were not completely separated, and the tailing of peaks was observed. Therefore, a system of ethyl acetate–methanol-33% ammonia solution was checked with different volumes of ammonia. It was discovered that separating MET from its impurity requires ammonia to be present in the developing system. However, the tailing still appeared, so glacial acetic acid and formic acid were also tried separately to solve the peak tailing. It has been shown that employing an acid in the developing system considerably improved the chromatographic isolation. The selection of the acid employed was optimal through a comparative analysis of glacial acetic acid and formic acid, while glacial acetic acid attained a preferable result. Then, the amount of glacial acetic acid was adjusted using various volumes (0.1–0.5 mL). An excellent separation with symmetrical peaks was achieved using 0.3 mL of acetic acid.

b) Wavelength

To accomplish good sensitivity with little noise, various scanning wavelengths, including (210, 215, 220, and 245 nm), were tested. The wavelength of 215 nm was determined to be the most suitable for scanning all components with satisfactory sensitivity and minimal noise-induced distortions in peak shapes.

c) Saturation time.

Several saturation times were assessed for the developed HPTLC method. The optimum developing system’s chromatographic tank time saturation was calculated and determined at 30 min.

**Fig. 1:**
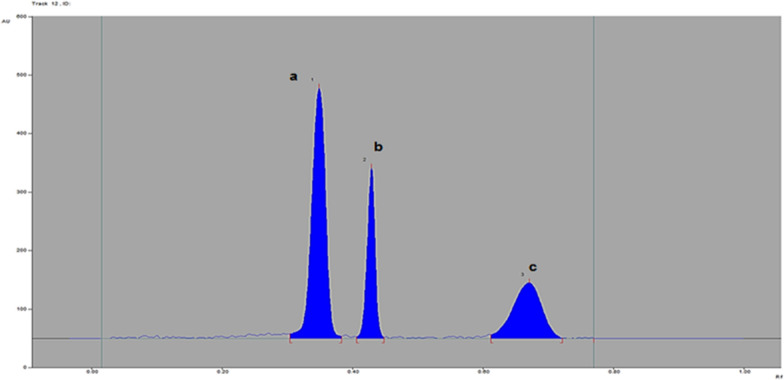
HPTLC-densitogram of a mixture of **a** metformin (0.5 μg/band), **b** Saxagliptin (0.5 μg/band), and **c** melamine (0.1 μg/band) using a developing system of ethyl acetate–methanol-ammonia-acetic acid (6:4:1:0.3, v/v/v/v)

As shown in Fig. 1, the R.F. values for SAX were 0.41, MET was 0.30, and MEL was 0.63 when created from a solution composed of ethyl acetate: methanol: ammonia: and glacial acetic acid (6:4:1:0.3, v/v/v/v). Detection was carried out at 215 nm.

#### UPLC method

In the context of this study, the objective is to achieve the isolation and quantification of the target compounds of SAX, MET, and MEL. An isocratic UPLC method with DAD detection was created. Without the intervention of excipients, the devised approach resolved the ternary combination in a single run utilizing the green mobile phase. All the previously published 13 HPLC techniques using various mobile phases failed to resolve the three components under investigation adequately. Upon reviewing the existing literature on chromatographic methods employed for quantifying SAX and MET as a combined combination, it was observed that methanol emerged as the preferred organic modifier. At the same time, the C18 column served as the stationary phase in these studies. The UPLC method showed advantages regarding time efficiency and selectivity since the separation process was completed within 5 min.

- Stationary phase

Various columns were utilized to optimize the stationary phase, including: C8 with dimensions of 25 cm × 4.6 mm i.d and a particle size of 5 μm, unfortunately did not yield optimal separation of SAX, and C18 with dimensions of 15 cm × 2.1 mm i.d and a particle size of 3 μm. The C18 column was found to be the ideal choice for separation, exhibiting minimal baseline noise and the shortest run time.

b) Mobile phase

Different mobile phases were investigated by varying proportions, considering system suitability parameters such as the tailing factor, number of theoretical plates, height equivalent to the theoretical plate, and retention time. Initial testing involved various mobile phases incorporating green solvents. First, ethanol and water were tried, but there was no successful separation of SAX, MET and MEL, pKa value of them 7.3, 12, 4 and 5 respectively adjusting pH was assessed using different basic and acidic pH values. Ethanol: water: orthophosphoric acid (H_3_ PO_4_) pH = 3, and the separation declined. After that, methanol and water were tried at different ratios: methanol: water: orthophosphoric acid (H_3_PO_4_) pH = 3 methanol: water: triethylamine (TEA) pH = 8. Inappropriately, there was no successful separation upon replacing water with different aqueous phase such as sodium dihydrogen phosphate (NaH_2_ PO_4_), pH = 4.5 (0.01 M) was employed as the aqueous phase, and organic solvents like methanol or ethanol were added for modification. Neither MET nor MEL were isolated, only SAX. Substituting NaH_2_PO_4_ with sodium dodecyl sulfate (SDS) (pH = 2.5 with H_3_PO_4_) Trials using 0.05, 0.07, and 0.01wv%, using 0.01% aqueous SDS showed that improved peak shape; trials using 0.01% SDS with ethanol produced good separation between MET and MEL but unacceptable separation between SAX and MET; and trials using methanol: sodium dodecyl sulfate (SDS) 0.01% (70:30, by volume), pH = 3.3with H_3_PO_4_) produced good separation. Two different pH levels (2.5 and 3.3) were also tested to determine their impact. Peak forms improved when the pH was adjusted to 3.3. After investigating with 1, 1.5, 2, and 2.5 mL/min flow rates, it was found that 1.5 mL/min provided the best resolution in a relatively quick (5-min) examination.

c) Wavelength

To improve the method's sensitivity, various detection wavelengths (210, 215,220, and 245 nm) were employed. The best data for the measured parameters were obtained when scanning at 215 nm, which had the highest sensitivity with minimum noise for the observed signals.

d) Temperature

The study employed a thermostatic column compartment to examine the impact of varying temperatures (20 °C, 25 °C, and 30 °C); subsequently, it was found that neither the chromatographic separation nor peak shape was impacted by the column temperature. So, the temperature value was retained at 25 °C during the analysis.

Subsequently, using a C_18_ column and methanol: 0.01% sodium dodecyl sulfate (pH of aqueous solution = 3.3 with orthophosphoric acid) (70:30, by volume) as the mobile phase, at a flow rate 1.5 mL/min, the chromatographic separation of SAX, MET, and MEL was completed. The temperature of the column was adjusted to 25 °C, and the detection process took place at a wavelength of 215 nm.

### Application of the developed methods

Once the procedures were optimized, they were utilized to ascertain the precise amounts of SAX and MET in pharmaceuticals, both in their most concentrated forms. Under the parameters of the chromatogram, the integrated peak area was found to be linearly related to the concentrations of SAX and MET.

### Method validation

The ICH guidelines [26] were used as a framework for the validation of the methodology.

#### Linearity

The HPTLC-densitometric technique demonstrated ranges of 0.2–10, 0.2–10, and 0.02–1 μg/band for SAX, MET, and MEL, separately. On the other hand, the UPLC method showed ranges of 0.5–100, 0.5–70, and 0.2–10 μg/mL for SAX, MET, and MEL, separately. These ranges were indicative of the linearity of the suggested methods for the HPTLC method. Table 1 presents the validation parameters that were computed for the proposed techniques.

**Table 1:** Regression and analytical parameters of the proposed methods for the determination of Saxagliptin, Metformin, and Melamine

Parameter	HPTLC–densitometric method		
	SAX	MET	MEL
Calibration range (μg/band)	0.2–10	0.2–10	0.02–1
Slope	0.0913	0.1789	1.0274
Intercept	0.0532	0.2126	0.061
Correlation coefficient (r)	0.999	0.9997	0.9999
Accuracy (mean %recovery)	100.59	101.53	99.77
Repeatability^a^ (%SD)	0.401	0.64	1.39
Intermediate precision^b^ (%SD)	1.27	1.44	1.76
LOD^c^ (μg/ mL)	0.05	0.05	0.005
LOQ^c^ (μg /mL)	0.17	0.16	0.018

UPLC method			
	SAX	MET	MEL
Calibration range (μg/mL)	(0.5–100)	(0.5–70)	(0.2–10)
Slope	0.0772	0.0145	0.7104
Intercept	4.6175	0.5828	1.2600
Correlation coefficient (r)	0.9999	0.9998	0.9999
Accuracy (mean %recovery)	101.28	101.36	100.26
Repeatability^a^ (%SD)	1.39	1.36	0.89
Intermediate precision^b^ (%SD)	1.48	1.62	1.13
LOD^c^ (μg/mL)	0.15	0.15	0.06
LOQ^c^ (μg/mL)	0.46	0.43	0.18

^a^The intra-day (n = 3), average of 3 different concentrations repeated 3 times daily

^b^The inter-day (n = 3), average of 3 different concentrations repeated 3 times in 3 successive days

^c^Limit of detection (3.3 × SD/slope) and limit of quantification (10 × SD/slope)

#### Accuracy

The accuracy of the proposed procedures was determined by calculating the percentage recoveries of pure samples of the examined components. The evaluation process involved the selection of five concentrations that encompassed the functional spectrums of the three components. The implemented methodologies showed favorable accuracy, as evidenced by the obtained results indicating typical percentage recoveries of 100.59%, 101.54%, and 99.77% for HPTLC analysis and 101.28%, 101.37%, and 100.26% for UPLC analysis, respectively, for SAX, MET, and MEL compounds, as presented in Table 1. The accuracy of Komiglyze e® X.R. pills was further verified using standard addition techniques. Table 2 exhibits the acquired percentage recoveries, which indicate the absence of any excipient impact.

**Table 2:** Results of analysis of Kombiglyze XR ® tablets and application of standard addition

Methods	HPTLC–densitometric method		UPLC method	
	SAX	MET	SAX	MET
KombiglyzeXR® tablets (B.N.W0046) %Founded ± RSD	102.92 ± 0.92	102.03 ± 0.99	99.18 ± 1.69	101.73 ± 1.97
n	6	6	6	6
Standard addition	102.52 ± 0.303	101.68 ± 0.19	100.77 ± 0.91	100.20 ± 1.96

#### Precision

The precision of the measurements was assessed using intra-day (repeatability) and inter-day (intermediate variances) tests. Standard solutions of 5, 10, and 15% for SAX, 2, 5, and 15%for MET, and 1, 2, and 3% for MEL in the HPTLC method were conducted from their respective stock solutions. In the UPLC method, standard solutions of 0.5, 5, and 10% for SAX, 0.5, 5, and 10% for MET, and 0.2, 0.5, and 1% for MEL were conducted from their respective working solutions. The intra-day Precision of each concentration was assessed using three tests on the same day. The Precision was quantified as the percent relative standard deviation (%RSD) with a sample size 9. The inter-day precision was determined by conducting tests on identical standard solutions (n = 9) over three consecutive days and subsequently determining the relative standard deviation (RSD). The obtained results of %RSD were found to be below 2%, indicating the high level of precision in the created method, as shown in Table 1

#### Limits of detection and quantification (LOD and LOQ)

Determining the limit of detection (LOD) and limit of quantification (LOQ) is crucial in assessing the sensitivity of the procedures. The formulae provided can be utilized to ascertain the limits of detection (LOD) and limits of quantification (LOQ) for SAX, MET, and MEL concentrations within the lower linear range of their respective calibration curves. Specifically, the LOD can be calculated using the formula LOD = 3.3 standard deviations (SD) divided by the slope. At the same time, the LOQ can be determined by dividing 10 times the SD by the slope, where SD is the standard deviation of the response variable. The data presented in Table 1 demonstrates the high level of sensitivity exhibited by the developed methodologies.

#### Specificity

**Fig. 2:**
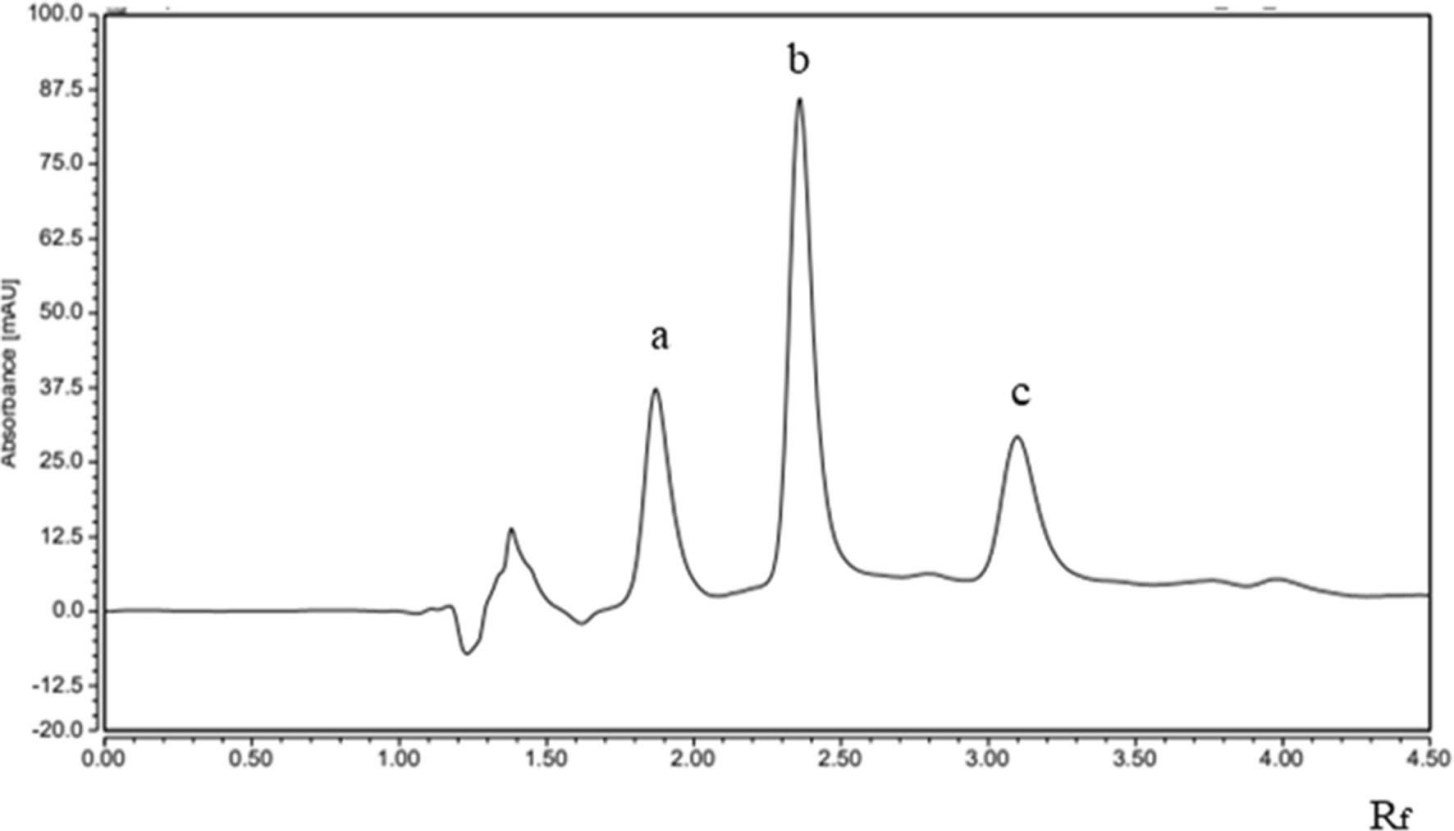
UPLC chromatogram of a mixture of **a** Saxagliptin (10 μg mL^–1^), **b** metformin (10 μg mL^–1^), and **c** melamine (1 μg mL^–1^) using a methanol–sodium lauryl sulfate (SDS) 0.01 m (70:30, by volume), pH = 3.3with H_3_PO_4_) as the mobile phase

The clear separation between the three researched components confirmed the distinctiveness of the methodologies employed, as depicted in Figs. 1, 2 and S.1, S.2, S.3 in supplementary file. Furthermore, the acquired % recoveries, found to be acceptable when the techniques were applied to the dosage forms currently available on the market, indicate that excipients did not cause any interference, as shown in Table 2. Method specificity as stability indicating was checked by applying to stability condition of studied drugs, SAX & MET were subjected separately to acidic hydrolysis (0.1N HCl), alkaline hydrolysis (0.1 N NaOH) and oxidative degradation (3% H_2_O_2_) for 8 h. The degradation solution for both drugs were applied to developed UPLC method, the peak area of MET was decreased in all. Degradation solution which refers to its susceptibility to degradation (align with the previous articles of MET stability study).But unfortunately, the degradation product were not well detected by the proposed UPLC, this is may due to their poor UV absorptivity at the working detection wavelength. Meanwhile, SAX was degraded under acidic & alkaline conditions (observed by decrease in it is peak area), but the degradation products interfere with the peak of melamine, as presented in S.4-S.6 in supplementary file.

#### Robustness

The performance of an analytical method is evaluated based on its robustness, which is determined by making deliberate and minor adjustments to the method parameters. The evaluation of conditions for the optimal utilization of procedures occurs during the creation and optimization of HPTLC and UPLC methods. The HPTLC-densitometry approach involved making slight modifications to the quantities of glacial acetic acid (± 0.1%), ammonia (± 1%), ethyl acetate (± 1%), methanol (± 1%), and the duration of saturation of the development chamber (± 5 min). The percentage relative standard deviation (%RSD) was calculated to determine the effect of these adjustments on the peak area in each case. In the meantime, the UHPLC technique was executed by the implementation of minor modifications in the mobile phase composition (± 1% deviation from the initial methanol composition), SDS concentration (± 1%), pH level (± 0.2), and flow rate (± 0.1 mL/min). The results in Table 3 illustrate the resilience of the developed methodologies and indicate that the examined variables did not influence the peak areas.

**Table 3:** Robustness results for the determination of Saxagliptin, Metformin, and Melamine by TLC–densitometric and UPLC methods

Parameters	Robustness [%RSD]		
	SAX	MET	MEL
HPTLC–densitometric method			
Composition of mobile phase ± 1% from the initial composition of Ethyl Acetate 6 ± 1%	1.42	1.56	1.34
Glacial acetic acid ratio 1 ± 1%	0.75	1.21	1.14
Ammonia ratio 0.3 ± 1%	0.58	0.67	0.43
Saturation Period 30 ± 5 min	0.85	0.65	1.19
UPLC method			
Change composition of mobile phase ± 1% from the initial composition of methanol 70 ± 1%	1.101	0.94	1.52
pH 3.3 ± 0.2	0.22	0.71	0.91
Flow rate 1.5 ± 0.1 mL/min	1.32	1.54	1.29

#### System suitability testing parameters

Following the system suitability testing, satisfactory outcomes were achieved, as indicated by the tailing factors, resolution (Rs) and selectivity factors (α) values over 1 and 1.5 respectively, these values ensured effective separation of each component from one another, as presented in Table 4. The obtained findings were within suitable limits [30].

**Table 4:** System suitability testing parameters of the developed TLC–densitometric and the UPLC methods

Parameters	HPTLC–densitometric method				UPLC method			Refer
	SAX	MET		MEL	SAX	MET	MEL	
Tailing factor (T)	1	0.90		0.99	1	1	1	< 1.5–2 or < 2
Capacity factor (K`)	–				1.025	1.55	2	1–10
Selectivity (α)	1.3			1.5	2.312		3.042	> 1
Resolution (R)	1.87			3.58	3		2.375	R > 1.5
Column efficiency (N) the separation	–				2916	25,550.25	3600	Increase with efficiency of the separation
HETP^a^	–				0.00093	0.00914	0.006	

^a^HETP: height equivalent to theoretical plate (cm per plate)

### Application to pharmaceutical formulation

The intention of this research was to test the analytical methods' suitability for usage in the analysis of commercially available pharmaceutical formulations by comparing their capacity to quantitatively detect SAX and MET in Kombiglyze® XR tablets. Excipients did not appear to cause any problems. The astounding recovery rates are displayed in Table 2. Studies of recovery using the refined standard addition procedures corroborated the validity of the proposed procedures.

### Statistical comparison with the reported method

The statistical study involved comparing the data obtained from the proposed methods for detecting SAX and MET in pharmaceutical formulation with the results obtained from a previously disclosed RP-HPLC method [17]. This comparison was conducted using the Student's t-test and the variance ratio F-test Table 5 confirms that there is no substantial discrepancy observed between the two sets of results**.**

**Table 5:** Statistical comparison of the results obtained by applying the proposed methods and the reported HPLC method for the determination of Saxagliptin and Metformin in pure form

Method	HPTLC–densitometric method		UPLC method		Reported method ^*^	
Items	SAX	MET	SAX	MET	SAX	MET
Mean	102.95	102.03	99.18	101.73	102.61	101.18
%RSD	0.92	0.99	1.69	1.97	1.69	0.94
Variance	3.71	0.98	2.88	3.88	2.87	0.88
n	6	6	6	6	6	6
Student’s t-test^**^	0.297	1.51	3.50	0.107	–	–
F-value^**^	1.29	1.11	1.004	4.39	–	–

^*^Reported HPLC method; Octadecylsilyl silica gel column (250 mm × 4 mm i.d., 5 μm particle size), mobile phase composed of methanol: water (30:70, v/v), flow rate: 0.5 mL/min, UV detection: 233 nm at 25 °C.The values of t and F tests represent the corresponding tabulated values of t and F at *p* = 0.05

^**^Student’s ttest = (2.228) F-test = (5.050)

### Pharmacokinetic prediction properties of melamine

One of the primary objectives of scientific research in the pharmaceutical industry is to find out the proportions and quantities of official impurities, as well as to predict the pharmacokinetic behavior within the body to elucidate its potential impact on human health. The pkCSM [31] software was employed in this investigation to forecast the toxicological characteristics of MEL, High-dosage melamine will result in urinary stones, crystal Luria, and acute renal failure. In addition, the human pharmacokinetic parameters of MEL, including absorption, distribution, metabolism, and excretion, were evaluated for the first time. The software evaluates multiple factors including water solubility, intestinal absorption, clearance, permeability across the blood–brain barrier, central nervous system effects, hepatotoxicity, and mutagenicity. The findings indicated that MEL exhibits limited water solubility and intestinal absorption, which might lower its bioavailability, exhibited inadequate permeability to the central nervous system and was unable to traverse the blood–brain barrier, hence diminishing the probability of inducing adverse effects in the brain, MEL can be considered as no CYP1A2 inhibitor so it may be involved no drug-drug interactions as well as no oxidative stress, NH2 group in melamine coupled with glucuronic acid, facilitating its excretion. The toxicological properties of MEL was also tasted, was found to be no hepatotoxic, Extremely toxic with maximum tolerated dose, it showed T. Pyriformis toxicity as detailed in Table 6. The paragraph highlights the necessity of creating accurate and specific analytical techniques to measure impurities in anti-diabetic drugs within their recognized renal toxic impurities. These procedures will ensure that impurities remain under acceptable standard limits.

**Table 6:** Pharmacokinetic prediction properties of melamine

Property	Items	Compound	Reference [30]
Absorption	Water solubility (log mol/L)	− 2.036	Solubility increased by decreasing log S
	CaCO-2 permeability (log Papp in 10^–6^ cm/S)	− 0.279	High permeability > 0.90
	Intestinal absorption (%)	61.225	High absorbed > 30%
	P-Glycoprotein substrate	No	
Distribution	VDss (log L/Kg)	− 0.497	Low < − 0.15
			High > 0.45
	Fraction unbound (Fu)	0.88	
	BBB permeability (log BB)	− 0.746	Log BB < − 1 poorly distributed to the brain
			Log BB > 0.3 cross the BBB
	CNS permeability (log PS)	− 3.549	Log PS < − 3 unable to penetrate CNS
			Log PS > − 2 penetrate CNS
Metabolism	CYP1A2 inhibitor	No	This can be positively correlated to the lipophilicity of the compound to metabolism related toxicity
	CYP2C19 inhibitor	No	
	CYP2C9 inhibitor	No	
Excretion	Total clearance (log mL/min/Kg)	0.071	
	Renal OCT2 substrate	No	
Toxicity	Max. tolerated dose (log mg/kg/day)	0.754	Low ≤ 0.477
			High > 0.477
	Oral rat acute toxicity (LD50) (mol/kg)	1.719	
	Hepatotoxicity	No	
	T. pyriformis toxicity (log µg/L)	− 0.222	Not toxic < − 0.5
			Toxic > − 0.5
	Minnow toxicity (log mM)	3.779	Highly acute toxic < − 0.3
			Not highly acute toxic > − 0.3

## Greenness assessment of the developed methods

The assessment of the environmental impact of the established methods involved the utilization of four distinct tools: the national environmental method index (NEMI), a qualitative approach; the analytical eco-scale method, a semi-quantitative approach; the green analytical procedure index (GAPI) tool, which provides comprehensive information on the evaluated processes; and the analytical greenness metric (AGREE). The AGREE pictogram comprises twelve components corresponding to the fundamental principles of green analytical chemistry (GAC).

### National environmental method index (NEMI)

The NEMI pictogram represents an early qualitative assessment tool [32] utilized for evaluating the environmental sustainability of a method. It employs a visually intuitive pictogram that offers immediate information and simplifies the understanding of the method's environmental impact. Just one look at the pictogram is adequate to learn about the method's environmental implications. The profile criteria, when summarized, consist of four fundamental terms. Methanol and ethyl acetate were not classified as persistent, bio accumulative, and toxic (PBT) compounds or as hazardous materials. They were found to possess a minimum quantity considered acceptable and safe for the utilization of ammonia and acetic acid.

Furthermore, the pH utilized in the experiment was non-corrosive, falling within the range of greater than 2 and less than 12. Additionally, the waste generated for each sample was less than 50 g. The HPTLC and UPLC method developed in this study is considered satisfactory, as it meets the acceptance criteria for all four quadrants, as presented in Table 7 and S.7 in supplementary file.

**Table 7:** Greenness assessment of the developed and reported chromatographic methods for the determination of Saxagliptin, Metformin and Melamine by NEMI, Analytical Eco-scale, GAPI and AGREE approaches

Analytical-Eco Scale	NEMI Pictogram	GAPI Assessment	AGREE
HPTLC–densitometric method			
Ethyl Acetate 4			
Methanol 6			
Acetic Acid 4			
Ammonia 6			
Energy 1			
Occupational 0 hazards			
Waste 3			
Total Penalty: 24			
Score: 76			
UPLC method			
Methanol 6			
SDS 0			
Phosphoric acid 2			
(PH = 3)			
Energy 1			
Occupational hazards 0			
Waste 3			
Total Penalty: 12 Score: 88			

### Analytical eco-scale assessments (ESA)

The predominant method utilized for generating a greenness profile is a semi-qualitative approach, whereby each parameter within the analytical procedure is assigned a penalty point (P.P.) [33–35]; the highest attainable eco-score for this method is 100. Postscript (P.P.S.) sections are included in the suggested process to address important aspects such as the quantity of reagents, potential occupational hazards, energy consumption, and trash generation. The specific eco-scale scoring for the suggested technique is presented in Table 7, providing further specifics. The HPTLC approach demonstrates a commendable total analytical eco-score of 76. At the same time, the UPLC method surpasses this threshold with an eco-score of 88, indicating its superiority in terms of environmental friendliness. Consequently, both methodologies may be classified as exemplary green analytical techniques.

### Green analytical procedure index (GAPI)

The green analytical procedure index (GAPI) [36] can be seen as a synthesis of the eco-scale and NEMI instruments. The environmental impact of each stage of an analytical process is thoroughly examined and quantified and is visually depicted by a distinct color-coded pictogram consisting of five pentagrams. The ecological ramifications of each stage, wherein green, yellow, and red signify minimal, moderate, and substantial consequences, respectively. Table 7 and S.8 in supplementary file presents GAPI pictograms illustrating the HPTLC, contains three red subsections 1, 13 and 15 these red sections refer to offline assay and five green subsections 2, 3, 4, 8 and 9 section refer to inline assay and contain seven yellow subsection 5, 7, 6, 10, 11, 12 and 14 refer to on line assay and UPLC Methods, contains three red subsections 1, 7, 15 these red section refer to offline assay and 5 green 2, 3, 4, 8, and 13 section refer to inline assay and contain seven subsection yellow 5, 6, 9, 10, 11, 12, 14 refer to online assay, wherein a greater proportion of green-shaded sections and a lesser proportion of red-shaded sections are observed.

### Analytical greenness metric (AGREE)

The software employed by the program has calculations that rely on 12 variables, each of which correlates with one of the 12 principles of green analytical chemistry [37]. A numerical value ranging from 0 to 1 is assigned to each principle or parameter, reflecting the risk associated with its environmental friendliness. The circular symbol emerges automatically as a representation of the ultimate assessment. The pictogram encompassed a range of 12 elements, including the instrument type, sample quantity, waste disposal, reagent type, and procedural steps. The findings of the AGREE pictogram and score were consistent with the previous results obtained from the HPTLC method (score of 0.77) and the UPLC method (score of 0.86), as presented in Table 7 and S.9 in supplementary file.

## Conclusion

The preliminary developments in HPTLC and UPLC techniques were instituted to assess the concentrations of Saxagliptin (SAX) and Metformin (MET) in the presence of a hazardous contaminant, melamine (MEL). The approach has several advantages, including rapid analysis timeframes, minimal sample pretreatment procedures, and high levels of precision and accuracy. Furthermore, they have been successfully employed to detect detrimental pollutants with sufficient sensitivity. Quality-control laboratories find them easy to utilize for drug analysis and impurity detection purposes.

## Supplementary Information

Supplementary Material 1.

## Data Availability

No datasets were generated or analysed during the current study.

## Abbreviations

SAX: Saxagliptin
MET: Metformin
MEL: Melarmine
HPTLC: High-performance thin layer chromatography
UPLC: Ultra-performance liquid chromatography
ESA: Analytical eco-scale assessments
AGREE: Analytical greenness metric
GAPI: Green analytical procedure index
SD: Standard deviations
LOD: Limits of detection
LOQ: Limits of quantification
ICH: International council for harmonization
