# Supplementary material for: Sequential separation of anti-diabetic drugs in the presence of melamine as impurity using chromatographic methods
Source: BMC Chem. 2025 Jan 28;19(1):26. doi: 10.1186/s13065-025-01385-6 (PMC11773764; doi:10.1186/s13065-025-01385-6)
Supplement: Supplementary file 1 — Supplementary Material 1. [file 13065_2025_1385_MOESM1_ESM.docx]

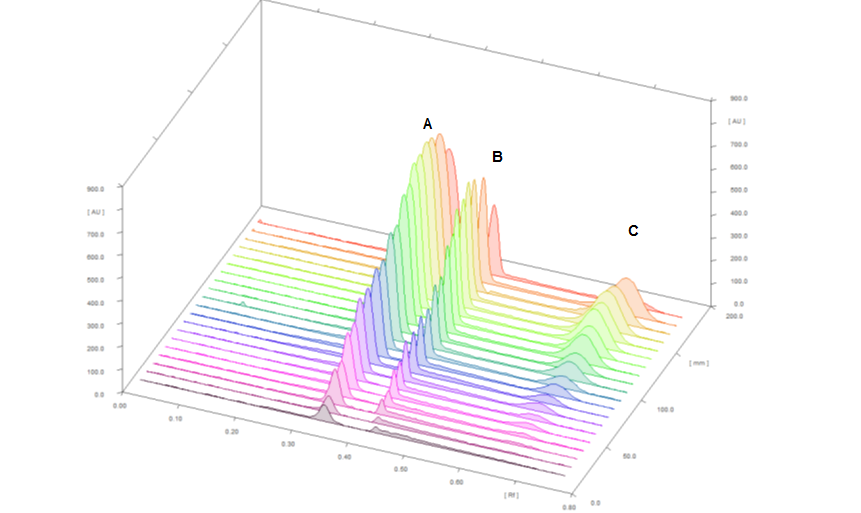


S. 1 . thin-layer chromatogram calibration of separated peaks of (A) metformin , (B) saxagliptin and (c) melamine using a developing system of ethyl acetate-methanol-ammonia-acetic acid (6:4:1:0.3, v/v/v/v).


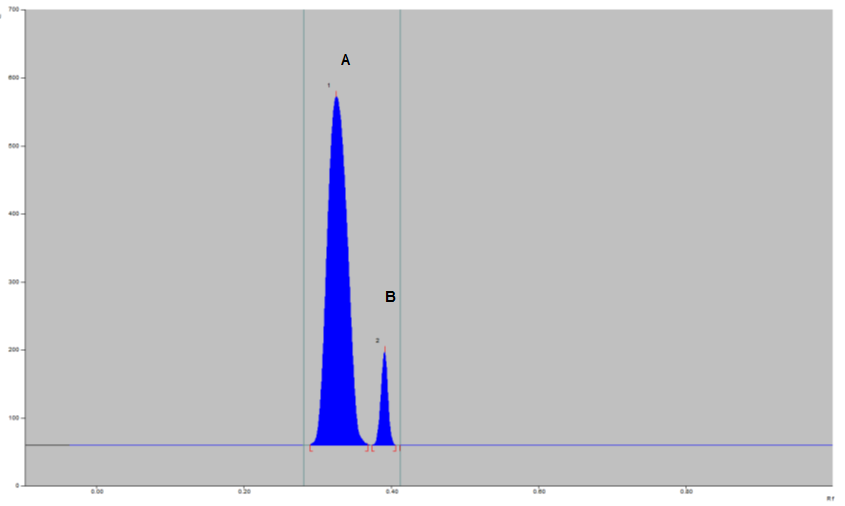


S.2 . thin layer chromatogram dosage form (A) metformin and (B) saxagliptin using a developing system of ethyl acetate-methanol-ammonia-acetic acid (6:4:1:0.3, v/v/v/v).


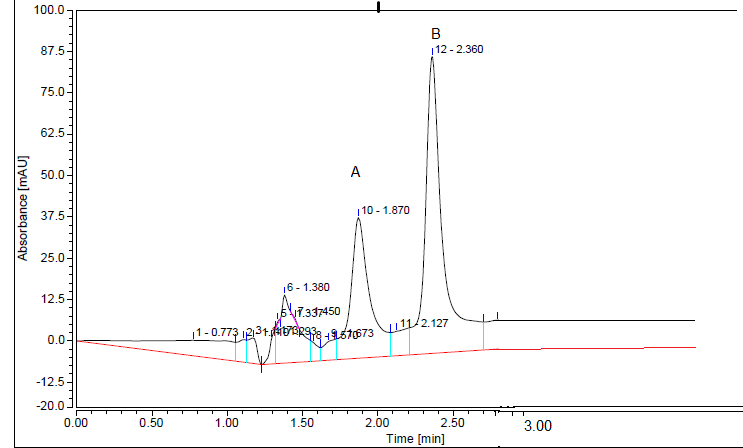


S. 3. Ultra- performance liquid chromatogram of a dosage form of (A) Saxagliptin, (B ) metformin using a methanol– Sodium lauryl sulfate (SDS)0.01 m(70:30, by volume), pH = 3.3with H3PO4) as the mobile phase .


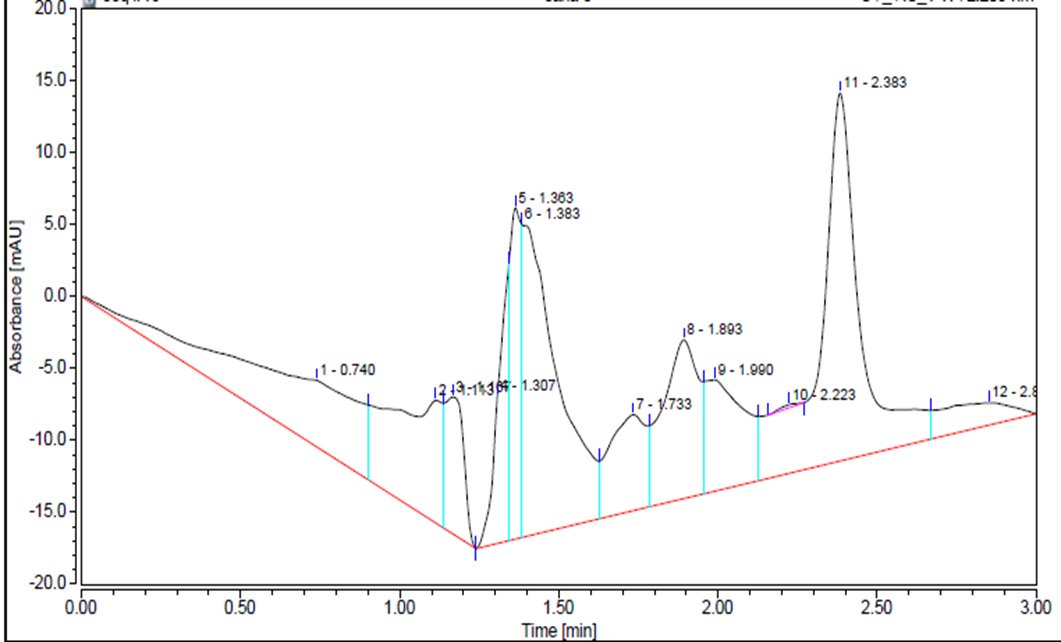
S.4. Hydrolytic degradation chromatogram of metformin under acidic ( 0.1N HCl) , alkaline (0.1N NaOH) conditions.


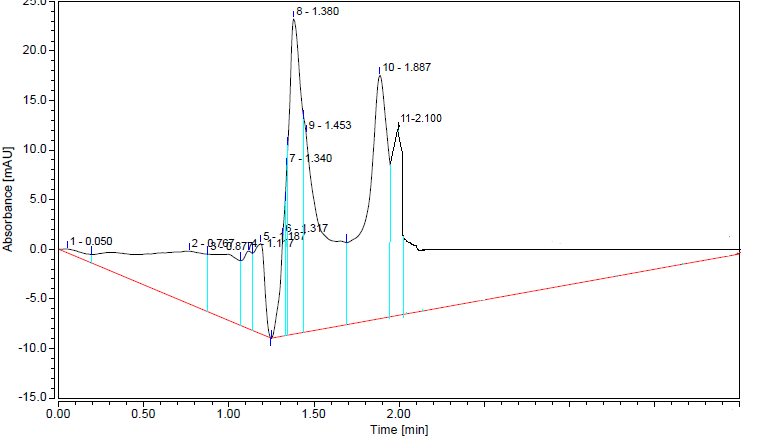


S.5. Hydrolytic degradation chromatogram of Saxagliptin under acidic (0.1N HCl) , alkaline (0.1N NaOH) conditions .


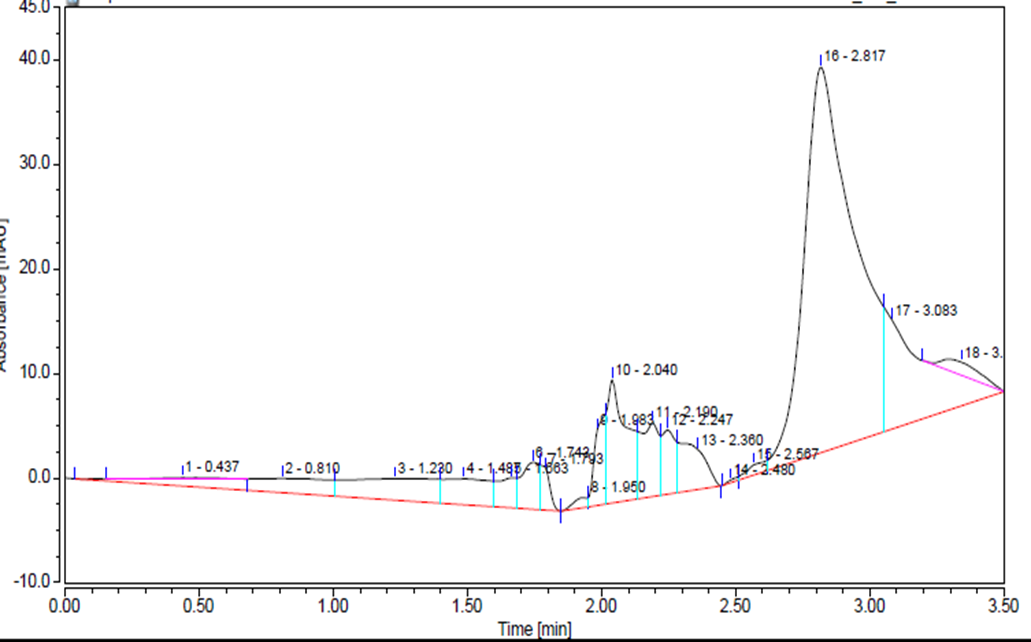


S.6.Oxidative degradation chromatogram of metformin under (3% H_2_O_2_) condition.


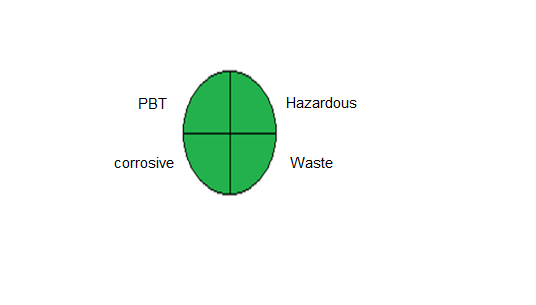


S. 7. The NEMI pictograms for assessment of “greenness” for HPTLC–densitometric method and UPLC method.


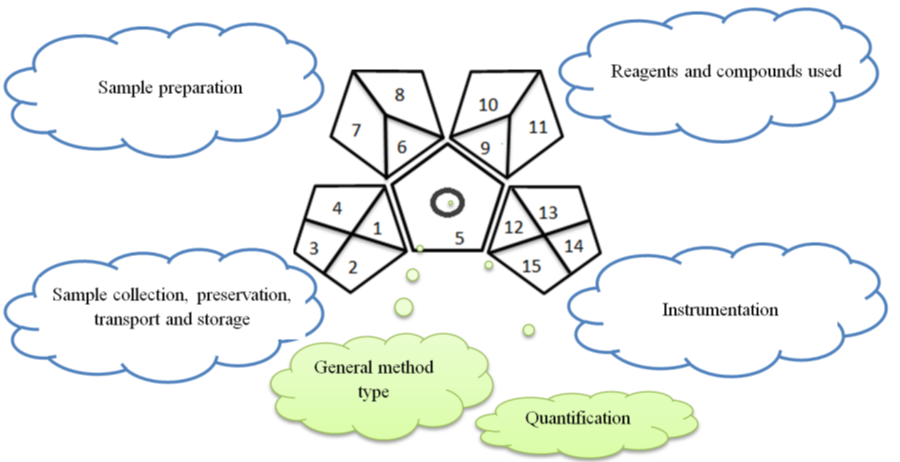


S. 8. Green Analytical Procedure Index pictogram with description.

| HPTLC–densitometric method | UPLC method |
| --- | --- |
| 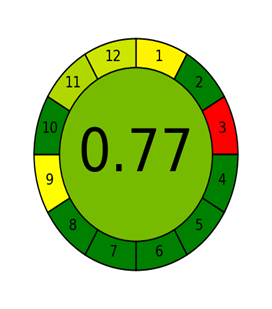 | 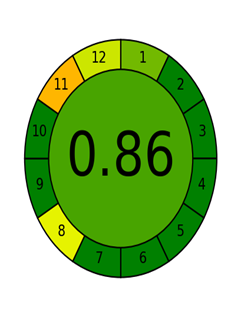 |
| 1. Sample treatment 2. Sample amount 3. Device positioning 4. Sample prep. stages 5. Automation, miniaturization 6. Derivatization 7. Waste 8. Analysis throughput 9. Energy consumption 10. Source of reagent 11. Toxicity 12. Operators safety | |

S.9.AGREE pictogram for assessment of “greenness” for HPTLC–densitometric method and UPLC method.
